# Mitochondrial Regulation of Microglial Immunometabolism in Alzheimer’s Disease

**DOI:** 10.3389/fimmu.2021.624538

**Published:** 2021-02-25

**Authors:** Lauren H. Fairley, Jia Hui Wong, Anna M. Barron

**Affiliations:** Neurobiology of Aging and Disease Laboratory, Lee Kong Chian School of Medicine, Nanyang Technological University Singapore, Singapore, Singapore

**Keywords:** beta amyloid (Aβ), neurodegeneration, metabolism, tau, microglia, mitochondria

## Abstract

Alzheimer’s disease (AD) is an age-associated terminal neurodegenerative disease with no effective treatments. Dysfunction of innate immunity is implicated in the pathogenesis of AD, with genetic studies supporting a causative role in the disease. Microglia, the effector cells of innate immunity in the brain, are highly plastic and perform a diverse range of specialist functions in AD, including phagocytosing and removing toxic aggregates of beta amyloid and tau that drive neurodegeneration. These immune functions require high energy demand, which is regulated by mitochondria. Reflecting this, microglia have been shown to be highly metabolically flexible, reprogramming their mitochondrial function upon inflammatory activation to meet their energy demands. However, AD-associated genetic risk factors and pathology impair microglial metabolic programming, and metabolic derailment has been shown to cause innate immune dysfunction in AD. These findings suggest that immunity and metabolic function are intricately linked processes, and targeting microglial metabolism offers a window of opportunity for therapeutic treatment of AD. Here, we review evidence for the role of metabolic programming in inflammatory functions in AD, and discuss mitochondrial-targeted immunotherapeutics for treatment of the disease.

## Introduction

Alzheimer’s disease (AD) is an age-associated terminal neurodegenerative disease characterized by the presence of two hallmark proteinopathies: extracellular aggregates of toxic beta amyloid (Aβ) and intracellular neuronal accumulation of misfolded tau. Inflammation is also an early feature of the neurodegenerative cascade (1, 2), identified as a key driver in pathogenesis and a promising target for AD therapeutic development (3). Although once viewed as a downstream consequence of AD pathogenesis, genome-wide association studies have implicated innate immunity as causative in the disease process, with variants of microglial regulators identified as key AD risk factors such as triggering receptor myeloid 2 (TREM2) and its downstream signaling molecule, Src homology 2 (SH2) domain containing inositol polyphosphate 5-phosphatase 1 (SHIP1) (4–8). Whether microglial overactivation or insufficiency contributes to neurodegeneration in AD is still a matter of controversy. However, in order to develop potential immunotherapeutics for AD, identification of mechanisms governing the switch between protective and dysfunctional microglial states is critical.

Emerging evidence indicates that metabolic function plays a critical role in the regulation of microglial immune function in AD, with several genetic risk factors for AD identified as important regulators of microglial metabolic fitness (3, 9, 10). Microglia are highly metabolically flexible and metabolic programming is a key regulator of functional plasticity, an emerging field known as immunometabolism (11, 12). Here we discuss the role of mitochondria as metabolic hubs and intracellular signaling platforms coordinating microglial immune functions in AD, and potential targets for the development of immunometabolic therapies for disease treatment.

## Innate Immunity and Metabolism in AD

Microglia perform a diverse range of specialist functions in the AD brain. They can mitigate neurodegeneration through phagocytic clearance of pathological Aβ (2, 13) and tau (14), by removing dying neurons thus preventing “bystander” neuronal death (15, 16) and releasing neurotrophic factors promoting neuronal support including nerve growth factor, brain-derived neurotrophic factor, and insulin-like growth factor-I (17–20). On the other hand, they can exacerbate neurodegeneration by mediating the spread of misfolded forms of tau, phagocytosing healthy or functional neurons, releasing neurotoxic cytokines and increasing oxidative stress through the generation of reactive oxygen species (ROS) (21–23).

Although activated microglia have previously been roughly divided into two categories—classically activated M1 host defence responses, with pro-inflammatory and cytotoxic properties, and alternatively activated M2 regeneration and repair responses—recent single cell transcriptomic analyses have revealed a high degree of heterogeneity and complexity within microglial states and populations that change with aging and disease (24–29). This microglial diversity is distinguished not only by unique immune signatures but also by altered metabolic phenotypes (3, 26, 29).

Diversity of mitochondrial structure, localization and function within and between cells has been identified as an important factor determining cell-to-cell heterogeneity, as well as contributing to heterogeneous outcomes in aging (30). In microglia, mitochondria coordinate energy supply, generation of ROS, and production of substrates for membrane biosynthesis, immune signaling molecules and growth factors (31). However, mitochondrial quality and activity declines in aging and age-related diseases, such as AD (32). Microglia have very low mitochondrial turnover (33) and are severely affected by mitochondrial impairment (34). Further, chronic exposure to pathogens such as Aβ and tau also induces mitochondrial toxicity and metabolic dysfunction in microglia (35). It is therefore important to understand the effect of aging and disease on microglial metabolic programming in AD.

## Microglial Metabolism in AD

Microglial metabolism is tightly controlled in response to environmental cues, including nutrient availability, cytokines, and damage- or pathogen-associated molecular patterns (DAMPs and PAMPs), including Aβ and tauopathy. A recent large-scale proteomics analysis of AD brain demonstrated early metabolic changes associated with microglial activation (3). Similarly, proteomics analysis of microglia isolated from AD mice identified enrichment in proteins involved in energy metabolism and mitochondrial processes (36). Here we will examine how the AD microenvironment impacts microglial metabolism and mitochondrial function.

### Nutrient Availability and Metabolic Stress

The AD brain is under metabolic stress, with impairments in nutrient availability including glucose occurring early in the disease due to impaired blood-brain glucose transfer (34, 37). Glucose is the primary substrate used by microglia for energy production and is taken into the cell *via* various glucose transporters (GLUTs) (38–40) where it can metabolise glucose *via* glycolysis in the cytoplasm, enzymatically converting glucose into pyruvate then lactate to generate energy in the form of adenosine triphosphate (ATP). Alternatively, pyruvate can be shuttled into the mitochondria where it is converted into acetyl coenzyme A (Acetyl CoA) and consumed by the tricarboxylic acid (TCA) cycle, generating substrates for oxidative phosphorylation (OXPHOS). Glycolysis is less efficient than OXPHOS, requiring nearly 20-fold more glucose to yield equivalent quantities of ATP, but much faster, making it ideal under conditions in which rapid energy production is required. Microglia express genes required for both glycolytic and oxidative metabolism (41, 42) and can switch between these metabolic programmes in response to inflammatory stimuli (35, 43–46).

Glucose can also be metabolized by microglia *via* the pentose phosphate pathway (PPP) to generate nicotinamide adenine dinucleotide phosphate (NADPH), which fuels NADPH oxidase to produce ROS as well as providing the building blocks for nucleotide synthesis (47). However, long-term reductions in glucose availability in aging and AD necessitate the use of alternative energy sources in microglia, or they risk metabolic derailment and dysfunction.

Microglia are also capable of using a range of non-glucose based energy sources, a potentially important adaptive function in the hypoglycemic AD brain. Fatty acids (FA), which are released following the degradation of lipid droplets, are transported into mitochondria and used to fuel mitochondrial OXPHOS, a process known as fatty acid β-oxidation (FAO). A number of genes involved in FAO are expressed in microglia (48), and FAs have been shown to fuel macrophage functions under glucose deprivation (49, 50). Microglia have also been shown to take up ketones and lactate, through the monocarboxylic transporters MCT1 and MCT2 (51). Glutamine is another alternative energy source consumed by microglia *via* glutaminolysis under hyperglycemic conditions, which feeds into the TCA cycle (52). Although the extent to which these non-glucose substrates fuel microglial function *in vivo* is largely unknown, a recent study used fluorescence lifetime imaging (FLIM) to indirectly measure glycolysis and OXPHOS in the normal mouse brain under insulin-induced hypoglycaemia, demonstrating microglia shift to glutaminolysis in the absence of glucose (52). Positron emission tomography (PET) can also be used to visualize glycolysis and OXPHOS in both animals and the humans. Glycolysis can be measured using the glucose analogue, ^18^F-fluoro-2-deoxy-D-glucose-PET (FDG-PET) (53, 54) in combination with cerebral oxygen consumption to measure glycolysis (55, 56), while OXPHOS can be measured using mitochondrial complex-I-PET (57). These metabolic imaging approaches will enable investigation of microglial metabolism in the living brain under both normal and AD conditions and may provide biomarkers of microglial metabolism.

### Aβ and Tau Alter Microglial Metabolism

AD-associated proteinopathy, Aβ and tau, induce mitochondrial toxicity and metabolic dysfunction. Exposure to Aβ alone or in combination with other inflammatory stimuli has been shown to induce a shift in metabolic programming from OXPHOS to glycolysis, impairing ATP production, increasing the generation of ROS, and inducing mitochondrial fission, fragmentation and extracellular release (35, 45, 46, 58). Fragmented mitochondria released from microglia have been shown to impair not only microglia, but also nearby neurons and astrocytes (58). Likewise, microglia isolated from AD mice are characterized by dependence on glycolytic metabolism as well as impaired mitochondrial quality control due to inhibition of mitophagy (59). Consistent with this, PET studies have shown increased reliance on aerobic glycolysis in areas spatially correlated with Aβ deposition in the AD brain (55, 56). Similarly, pathological forms of tau can bind mitochondria and impair OXPHOS and ATP synthesis (60); and Aβ and tau act synergistically to induce defects in OXPHOS and ATP synthesis, while increasing ROS production in AD mice (61).

Aβ can influence mitochondrial metabolism through direct binding and accumulation within mitochondria (62–64), inducing toxicity (65–67) and mitochondrial bioenergetic impairments (68–70). Mechanistically, Aβ inhibits OXPHOS by targeting ATP synthase, the enzyme that catalyses ATP production in the final step of OXPHOS (71). Others have implicated the nutrient sensor, mammalian target of rapamycin (mTOR), in Aβ-induced microglial metabolic reprogramming. Exposure to Aβ increases phosphorylation of mTOR *via* serine/threonine protein kinase B (Akt), which increases the expression of HIF-1a, the master transcriptional regulator of glycolysis (35).

Additionally, Aβ and tau can indirectly affect metabolism through upregulation of cytokines classically associated with M1, proinflammatory responses, such as interleukin-1β (IL-1β) and interferon-γ (IFNγ). Inflammatory cytokines induce glycolytic programming accompanied by breaks in the TCA cycle and uncoupling of OXPHOS (43, 45, 46). OXPHOS uncoupling impairs ATP production from mitochondrial respiratory chain activity, causing an increase in ROS generation (72). To support glycolysis in response to pro-inflammatory cytokines, microglia upregulate expression of GLUTs to facilitate increased glucose (40). A metabolic break in citrate metabolism fragments the TCA cycle, increases citrate availability for FA synthesis (FAS) and lipogenesis (73). Further, Aβ has been implicated in the suppression of mitochondrial succinate dehydrogenase (74), which underlies a second TCA cycle break widely observed in pro-inflammatory microglia, leading to accumulation of succinate. These findings suggest that AD-associated stimuli directly alter metabolic processes in microglia in a variety of ways. However, the precise signaling mechanisms involved remain to be fully elucidated.

### AD Genetic Risk Factors and Microglial Metabolism

A number of immune-related genetic risk factors for AD have been shown to modulate microglial metabolism. The most prevalent genetic risk factor for AD, apolipoprotein E4 (ApoE4), is primarily expressed by glia in the brain and has been shown to play a role in mitochondrial energy production (9). In human iPSC-derived microglia, the AD-associated E4 variant of ApoE severely impaired metabolism, inhibiting both glycolysis and OXPHOS (75). Further, cognitively normal ApoE4 carriers demonstrate abnormally low cerebral metabolic rates for glucose (76). Additionally, ApoE binds and transports lipoproteins, and ApoE knockdown has been shown to alter FA levels and lipid metabolism in the brain (77). Interestingly, ApoE is a ligand for TREM2, which is a microglial surface receptor required for diverse microglial responses in neurodegeneration, including proliferation, survival, clustering and phagocytosis (46, 78–80). Increased risk of developing AD is associated with loss-of-function variants of TREM2, and TREM2 has been shown to induce ApoE signaling in microglia (27, 81).

Recently, TREM2 has also been identified as a regulator of mitochondrial metabolic fitness in microglia and macrophages (10). TREM2-deficient microglia exhibit decreased expression of genes encoding glucose transporters, glycolytic enzymes, as well as decreased expression of the metabolic coordinator, mTOR (10). Combined transcriptomic and metabolic analysis of TREM2 deficient macrophages revealed reduced ATP production and defects in metabolites and enzymes involved in glycolysis, the TCA cycle and PPP (10). Furthermore, TREM2 knockout mice exhibit cerebral hypometabolism measured by FDG-PET (82, 83). TREM2 also plays an important role in lipid metabolism, with TREM2 deficiency causing pathogenic lipid accumulation in microglia (84).

The TREM2/ApoE axis has also been identified as a regulatory checkpoint in the differentiation of specialized microglial phenotypes associated with age and disease, coined disease-associated microglia (DAMs) (26). DAMs express high levels of lipid metabolism genes, including APOE, Cst7 and lipoprotein lipase (Lpl), which catalyse the release of FAs for FAO (50). Other studies have identified disease and injury associated microglial signatures that share overlap with some key DAM-associated genes, including APOE and Lpl (24, 25, 27). A recent study of microglial proteomic changes in AD mice demonstrated upregulation of the TREM2/ApoE axis and increased proteins involved in FA metabolism (85).

**Figure 1:**
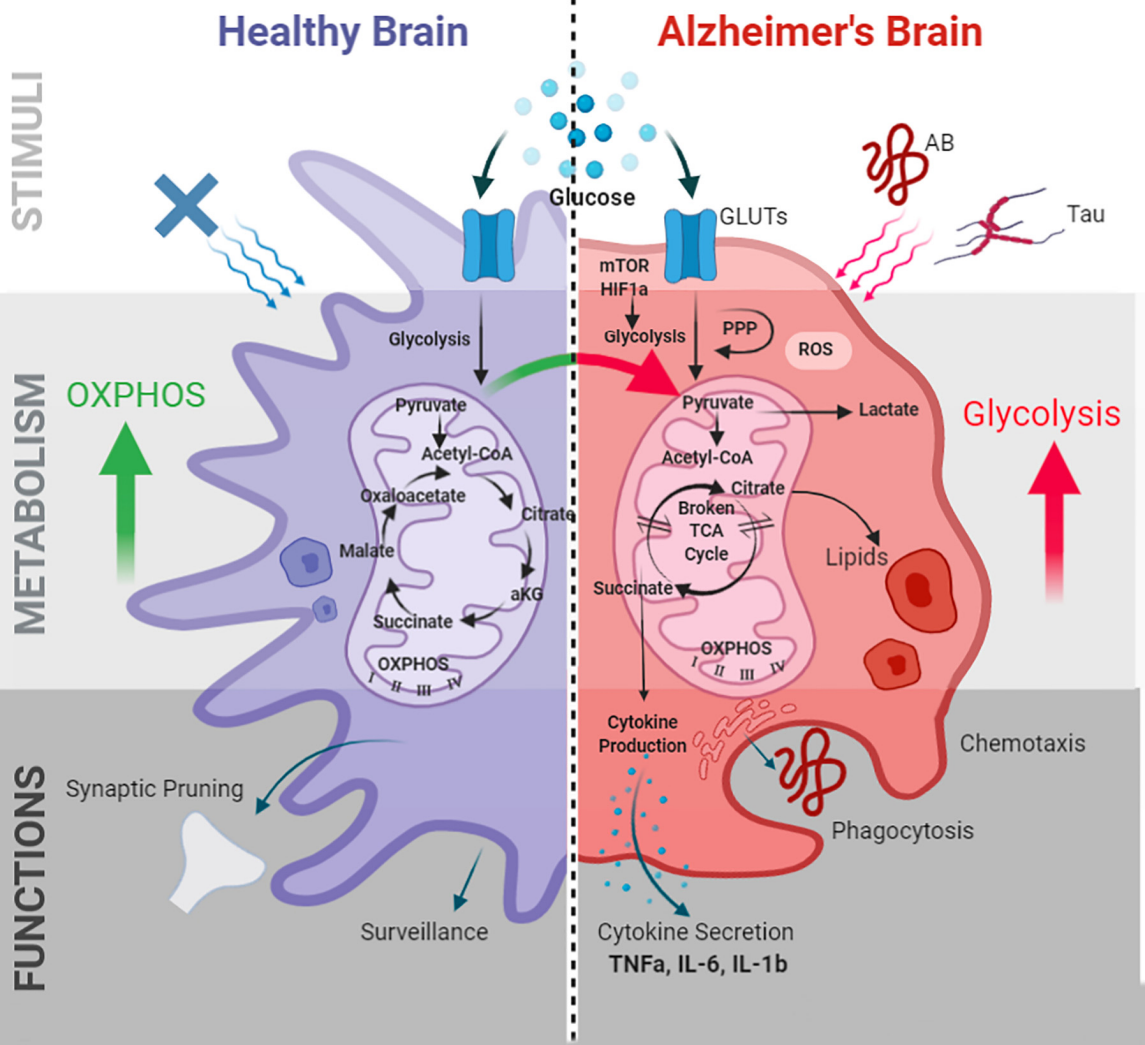
Microglial metabolic programming and immune functions in AD. Alzheimer’s pathogenic stimuli Aβ and tau induce microglial metabolic alterations. Metabolic alterations are mediated by the mTOR-HIF1a pathway and characterized by decreased OXPHOS, increased glycolysis, impaired ATP production, a “broken” TCA cycle, increased ROS, and lipid droplet accumulation. These alterations in turn effect microglial immune functions including phagocytosis, chemotaxis, cytokine production, membrane biogenesis, and antigen presentation. GLUTs, glucose transporters; PPP, pentose phosphate pathway; TCA, tricarboxylic acid; ROS, reactive oxygen species; OXPHOS, oxidative phosphorylation; TNF-α, tumor necrosis factor- α; IL-6, interleukin-6; IL-1β, interleukin-1β; HIF-1α, hypoxia inducible factor-1α; mTOR, mammalian target of rapamycin.

These findings indicate that AD-associated pathology and genetic risk factors are intricately associated with mitochondrial metabolic functions in microglia (summarized in **Figure 1**). However, further research is needed to elucidate the mechanistic pathways involved in metabolic regulation in microglia, which may in turn aid the identification of druggable targets to modulate immunometabolic impairment.

### Aging and Microglial Metabolism

Aging is associated with a glycolytic metabolic shift in both human and mouse microglia, coupled with increased expression of markers of cellular senescence (86, 87). Aging also leads to accumulation of lipid droplets in microglia in the mouse and human brain, named lipid droplet accumulating microglia (LDAM) (29). LDAMs are characterized by upregulation of genes involved in lipogenesis, TCA cycle and FAO, and exhibit increased ROS production. Meanwhile, key enzymes involved in lipid degradation are downregulated. A recent study has demonstrated that physical coupling between the mitochondria and lipid droplets plays an important role in the regulation of glycolytic metabolic reprogramming in immune cells (88). Whether lipid droplet-mitochondrial interactions regulate metabolic programming in microglia remains to be addressed, but may provide important new insights into how microglial metabolism is coordinated in aging and AD.

## Microglial Metabolism and the Innate Immune Response in AD

Microglial metabolism and immune function are reciprocally regulated. Microglia not only undergo adaptive metabolic reprogramming in response to inflammatory stimuli, but immune responses are dependent upon these metabolic shifts. Changes in cell morphology, chemotaxis, and phagocytosis all require reorganization of the actin cytoskeleton, which is dependent on the coordinated supply of ATP (89). Phagocytic degradation of engulfed materials, which plays an important role in the clearance of Aβ and tau, also relies upon the coordinated production and delivery of mitochondrial ROS to the phagolysosome (90). Similarly, the production of cytokines and growth factors requires resources such as amino acids, nucleotides, and fatty acids, which are supplied by metabolites generated during energy production. Mitochondrial metabolites are also utilized for lipogenesis to support membrane biogenesis for filopodia formation, antigen presentation and organelle biogenesis during proliferation and growth (73, 91). As such, changes in microglial adaptive metabolic reprogramming underpins immune function. Here we will discuss how age and disease associated changes in microglial metabolic programming modulates critical microglial functions in AD.

In microglia, initiation of the classic pro-inflammatory response is dependent upon glycolytic metabolic reprogramming, with immune responses including phagocytosis and pro-inflammatory cytokine production blocked by inhibition of glycolysis (25, 40, 92–94). In addition to rapidly generating ATP for energetically demanding chemotaxis and phagocytosis, upregulation of glycolysis and its branched pathway, the PPP, has been shown to be essential for the production of ROS-dependent phagosome degradation (95). In the AD brain, proteomics analysis has demonstrated a strong association between microglia and glycolytic metabolism (3). Upregulation of markers enriched in the AD brain was observed in microglia undergoing active Aβ phagocytosis in AD mouse brain, suggesting a protective function of hyperglycolytic microglia in AD (3). Markers identified in this study overlapped with markers of the TREM2-dependent, protective, phagocytic microglial subpopulation, DAMs (26). Knockout studies have shown that TREM2 promotes a metabolic programme fuelled by glycolysis, PPP and the TCA cycle to support phagocytosis (10). TREM2 deletion impairs microglial chemotaxis and phagocytosis in AD mice, resulting in inability of microglia to cluster around and clear aggregates of Aβ (10). These microglial deficits were mitigated with dietary cyclocreatine, a creatine analog that can supply ATP, indicating metabolic impairments caused the immune function impairments following TREM2 deletion (10).

Microglial hyperglycolysis is also observed in the aging brain but, in contrast is associated with compromised chemotaxis, phagocytosis and Aβ engulfment, and elevated secretion of pro-inflammatory cytokines (86, 87). Likewise, Aβ-induced glycolysis is associated with impaired chemotaxis and phagocytosis of Aβ in cultured microglia, a phenomena also observed in microglia isolated from AD mice (59). Further, multiple studies have shown that microglial Aβ phagocytosis is enhanced by promoting OXPHOS, rather than glycolysis (96, 97). One potential explanation for these differences may come from the chronic versus acute effects of Aβ on microglial function. Because glycolysis is metabolically inefficient, persistent reliance on glycolysis in microglia may lead to impaired immune function and reduced capacity to perform immune functions over time (97). In line with this, acute exposure to Aβ increased glycolysis and enhanced immune functions in microglia, whereas chronic exposure induced metabolic dysregulation and diminished immune functions, including phagocytosis and cytokine secretion (35). Further, Aβ-induced mitochondrial toxicity may disrupt coordinated immunometabolic programming. Consistent with this, in AD mice pharmacological induction of mitophagy to restore mitochondrial quality control enhanced microglial phagocytosis of Aβ and decreased the production of pro-inflammatory cytokines TNF-α and IL-6 (98).

Lipid metabolism has also been identified as important in microglial phagocytic functions in aging and AD. Lpl, the major enzyme responsible for liberating FAs from lipid droplets, is upregulated by Aβ in microglia, and silencing Lpl has been shown to impair Aβ phagocytosis (99). In macrophages, phagocytosis has been shown to be dependent on the availability of FAs following the degradation of lipid droplets, linking effective lysis of lipid droplets with successful phagocytosis (49). Supporting this, Lpl has been identified as a key marker of DAMs. In contrast, LDAMs, which are characterized by lipid droplet accumulation, lipogenesis and reduced FAO, also exhibit impaired phagocytosis (29). Lipid accumulation is a key feature observed in immune dysfunction, for example foamy macrophages observed in atherosclerotic lesions and lipid droplets have been identified as potential structural markers of inflammation (100). Interestingly, inhibition of FA synthesis restored phagocytic function in these microglia, again highlighting the potential to restore microglial immune function by restoring metabolic function in aging and AD.

## Microglial Metabolic Reprogramming for the Treatment of AD

AD is one of the leading causes of death worldwide with no effective treatments available, leading to urgent calls for the development of disease modifying-agents (101). Given the pivotal role of inflammation in AD pathophysiology, here we discuss potential therapeutic strategies that improve microglial function through regulation of metabolism.

### Ketone Body Therapeutics

Microglia can utilize ketone bodies as an alternative energy substrate to glucose, and ketosis has been shown to modulate a range of microglial inflammatory processes and reduce Aβ and tau accumulation in AD mice (101–106). Ketosis can be induced through several methods, including dietary modification, ketone body supplements, and pharmacological inhibitors of glycolysis. High-fat, low-carbohydrate ketogenic diets are thought to trigger a shift from glucose metabolism towards fatty acid metabolism, which in turn yields increased ketone body concentrations. Ketogenic diet decreased microglia activation and pro-inflammatory cytokine IL-6, IL-1β and TNF-α levels (107). Similarly, oral administration of ketone body metabolites such as β-hydroxybutyrate (β-OHB) have been shown to reduce microglial inflammation (108), reduce expression of pro-inflammatory cytokines IL-1b, IL-6, CCL2/MCP-1 (109), and inhibit NLRP3 inflammasome activation (110). Competitive inhibitors of glycolysis, such as 2-deoxy-D-glucose (2-DG) have been shown to induce compensatory metabolic processes and promote ketosis. Transgenic AD mice fed a diet supplemented with 2-DG exhibited increased serum ketone body levels and brain expression of enzymes required for ketone body metabolism, as well as decreased oxidative stress and reduced levels of Aβ oligomers (111). Further, pharmacological treatment with 2-DG has been shown to reduce markers of microglial activation following LPS treatment (41), reduce expression of inducible nitric oxide synthase (iNOS) (112), and decrease IL-6, IL-1β levels (113) in BV2 and primary microglia. These findings suggest that treatments targeted towards increasing microglial ketosis in AD may have therapeutic benefits, however ketogenic diet, β-OHB, and 2-DG are all known to exert non-microglial specific effects in a range of cell types in the brain. Further research aimed at identifying microglia-specific promoters of ketosis may be of benefit in the treatment of neuroinflammatory diseases such as AD.

### Exercise

Exercise is consistently associated with improvement in cognitive and neuronal function in aged animals (114, 115) and reductions in Aβ and tau pathology in AD mice (116–118). The mechanism underlying exercise-related benefits in the brain is not well understood, however decreased pro-inflammatory cytokine expression has been proposed as one potential mechanism (119, 120). Recently, metabolic reprogramming has been identified as a mediator of exercise-relate changes in cognition and immune functions, as exercise attenuated age-dependent inflammatory cytokine expression and cognitive decline in mice, while decreasing glycolytic enzymes and increasing phagocytosis in isolated microglia (86). However, whether exercise exerts beneficial effects by promoting OXPHOS or FAO, or alternative substrate use, remains to be elucidated. Although no study to date has investigated whether metabolic reprogramming underlies exercise-related changes in inflammation and pathology in an AD-specific context, these findings suggest that exercise is a promising avenue for therapeutic investigation.

### mTOR Targeted Therapeutics

The mTOR-HIF-1α pathway is a central mediator of inflammation in the brain and has been implicated in the regulation of microglial metabolic reprogramming in AD (10, 35). Two compounds that target the mTOR pathway and are currently being trialled for clinical efficacy in AD are rapamycin and metformin. Rapamycin directly inhibits mTOR *via* binding of mTOR Complex 1 (mTORC1), whereas metformin acts upstream of mTOR by targeting the glycolytic inhibitor AMP-activated protein kinase (AMPK). Both compounds have been shown to reduce glycolytic metabolism in favor of increased OXPHOS in immune cells (121, 122) and decrease the production of proinflammatory cytokines by microglia and macrophages (123, 124). As the mTOR pathway is found ubiquitously in all cell types, targeting microglial-specific mTOR signaling is challenging and both rapamycin and metformin are known to exert non-immune related effects in neurons and astrocytes. TREM2 has recently been identified as a microglial-specific target that regulates mTOR in AD (10), however there is currently a paucity of druggable targets identified in the TREM2-mTOR signaling pathway. SHIP1, which is primarily expressed in microglia, is one of the only therapeutic targets in the TREM2-specific mTOR signaling pathway that is under investigation for the treatment of AD. SHIP1 is known to inhibit TREM2 signaling (125) and pan-SHIP1/2 inhibitors have been shown to increase microglial phagocytosis of Aβ1-42 *in vitro via* mTOR regulation (126). These findings suggest that modulating TREM2-dependent mTOR signaling could provide neuroprotective effects in AD, however further research is needed to identify additional targets for therapeutic modulation in this pathway.

### TSPO Targeted Therapeutics

The translocator protein (TSPO) is an outer mitochondrial membrane protein that is predominantly expressed in microglia in the brain and is upregulated in AD (127, 128). Consequently, TSPO is widely regarded as a biomarker of neuroinflammation, and TSPO ligands have been shown to exert a range of protective effects in mouse models of neurodegeneration (129, 130). In particular, ligands targeting TSPO have been shown to decrease Aβ deposition and reduce markers of inflammation in mouse models of AD (131). TSPO has also been implicated in microglial metabolic programming, as TSPO deficiency suppressed both OXPHOS and glycolysis, resulting in overall metabolic deficits in primary microglia (132) and increased fatty acid oxidation in steroidogenic cells (133). In contrast, treatment with TSPO ligands improved mitochondrial respiration, decreased oxidative stress-induced cell death by reducing ROS, and lowered Aβ levels in H1299 cells (134). These findings suggest that TSPO may be a marker of beneficial microglial phenotypes, and treatments aimed at increasing TSPO expression may confer neuroprotective effects in AD. However, further research investigating the metabolic modulatory effects of TSPO ligands in microglia specifically is required.

## Discussion

Innate immunity plays a causative role in the pathogenesis of AD, and coordinated microglial immune responses have the potential to either ameliorate or exacerbate AD pathology, depending on microglial phenotypes. However, efforts to elucidate the cellular mechanisms and molecular signals mediating neuroinflammatory responses in AD have been hampered by the large heterogeneity in immune cell types and responses within the brain. Emerging evidence indicates that metabolic function plays a critical role in the regulation of microglial function in AD, with metabolic programming underlying diverse microglial immune functions and phenotypes. Further, therapeutic strategies modulating microglial metabolic programming have shown neuroprotective effects, by reducing amyloid and tau load, and improving cognitive deficits. These findings suggest that microglial function and metabolism are intricately associated processes in AD, however, further research is needed to elucidate the mechanistic pathways involved in metabolic regulation in microglia, which may in turn aid in the identification of druggable targets to modulate immunometabolic impairment.

## Author Contributions

LF, JW, and AB wrote and reviewed the manuscript. LF: prepared the figure. All authors contributed to the article and approved the submitted version.

## Funding

AB acknowledges funding support from the Singapore Ministry of Education under its Singapore Ministry of Education Academic Research Fund Tier 1 (RG42/18), Nanyang Assistant Professorship from Nanyang Technological University Singapore, and the Alzheimer’s Association (AARG-18-566427).

## Conflict of Interest

The authors declare that the research was conducted in the absence of any commercial or financial relationships that could be construed as a potential conflict of interest.
